# Acute *Penicillium marneffei* infection stimulates host M1/M2a macrophages polarization in BALB/C mice

**DOI:** 10.1186/s12866-017-1086-3

**Published:** 2017-08-18

**Authors:** Xiaoying Dai, Congzheng Mao, Xiuwan Lan, Huan Chen, Meihua Li, Jing Bai, Jingmin Deng, Qiuli Liang, Jianquan Zhang, Xiaoning Zhong, Yi Liang, Jiangtao Fan, Honglin Luo, Zhiyi He

**Affiliations:** 1 Department of Respiratory Medicine, The First Affiliated Hospital of Guangxi Medical University, Nanning, Guangxi 530021 China; 2 Guangxi Colleges and Universities Key Laboratory of Preclinical Medicine Research, Nanning, Guangxi 530021 China; 3 Department of Gynecology, The First Affiliated Hospital of Guangxi Medical University, Nanning, Guangxi 530021 China; 4 Guangxi Medical University, Nanning, Guangxi 530021 China

**Keywords:** *Penicillium marneffei*, Immune response, Alveolar macrophages polarization, CYA

## Abstract

**Background:**

*Penicillium marneffei* (*P. marneffei*) is a thermally dimorphic fungus pathogen that causes fatal infection. Alveolar macrophages are innate immune cells that have critical roles in protection against pulmonary fungal pathogens and the macrophage polarization state has the potential to be a deciding factor in disease progression or resolution. The aim of this study was to investigate mouse alveolar macrophage polarization states during *P. marneffei* infection.

**Results:**

We used enzyme-linked immunosorbent (ELISA) assays, quantitative real-time PCR (qRT-PCR), and Griess, arginase activity to evaluate the phenotypic markers of alveolar macrophages from BALB/C mice infected with *P. marneffei*. We then treated alveolar macrophages from infected mice with *P. marneffei* cytoplasmic yeast antigen (CYA) and investigated alveolar macrophage phenotypic markers in order to identify macrophage polarization in response to *P. marneffei* antigens. Our results showed: i) *P. marneffei* infection significantly enhanced the expression of classically activated macrophage (M1)-phenotypic markers (inducible nitric oxide synthase [iNOS] mRNA, nitric oxide [NO], interleukin-12 [IL-12], tumor necrosis factor-alpha [TNF-α]) and alternatively activated macrophage (M2a)-phenotypic markers (arginase1 [Arg1] mRNA, urea) during the second week post-infection. This significantly decreased during the fourth week post-infection. ii) During *P. marneffei* infection, CYA stimulation also significantly enhanced the expression of M1 and M2a-phenotypic markers, consistent with the results for *P*. *marneffei* infection and CYA stimulation preferentially induced M1 subtype.

**Conclusions:**

The data from the current study demonstrated that alveolar macrophage M1/M2a subtypes were present in host defense against acute *P*. *marneffei* infection and that CYA could mimic *P*. *marneffei* to induce a host immune response with enhanced M1 subtype. This could be useful for investigating the enhancement of host anti-*P*. *marneffei* immune responses and to provide novel ideas for prevention of *P*. *marneffei*-infection.

**Electronic supplementary material:**

The online version of this article (doi:10.1186/s12866-017-1086-3) contains supplementary material, which is available to authorized users.

## Background

*Penicillium marneffei* (*P. marneffei*)*,* discovered in 1956 by Capponi et al., is a thermally dimorphic *Penicillium* that causes a lethal systemic infection, even though other *Penicillium* species are usually not pathogenic to humans [1]. Clinically, *P. marneffei* is one of the most important opportunistic infectious pathogens in Southeast Asia and can cause a life-threatening systemic mycosis in immunocompromised individuals [2], and sometimes in immunocompetent individuals [3, 4]. For example, Hu et al. [5] reviewed 668 cases of *P*. *marneffei* between 1984 and December 2009 in Mainland China and showed that 99.4% of cases were reported in the southern part of China (Guangxi and Guangdong provinces), with 87.7% of these infections occurring in patients with human immunodeficiency virus (HIV). Only 8.5% of patients did not have HIV. HIV-reduced levels of CD4^+^ T cells could make host anti-*P*. *marneffei* immune responses ineffective, and therefore, make *P*. *marneffei* infection difficult to control in such patients. Therefore, HIV patients successfully treated for *P*. *marneffei* infection should receive long-term maintenance therapy to prevent recurrence [6]. Most opportunistic infections, such as *Cryptococcus neoformans*, *Aspergillus fumigatus*, and *Pneumocystis*, rely more heavily on innate immunity when they are T cell deficient. Macrophages are innate immune cells that have critical roles in protection against pulmonary fungal pathogens, including *C. neoformans*, *A. fumigatus*, *Pneumocystis* and *Candida albicans* [7–10]. Macrophage polarization state has the potential to be a deciding factor in disease progression or resolution [11].

In the environment, *P*. *marneffei* can grow at 25 °C as filaments and at 37 °C as yeast, and cause fatal disseminated and systemic infection in humans or rodent hosts, such as the bamboo rat [12, 13]. Clinically, *P*. *marneffei* infection usually appears in the lung, which could be due to inhalation of airborne *P*. *marneffei* conidia [14]. Furthermore, *P*. *marneffei* conidia are small enough to reach the alveolar spaces [14], leading to activation of alveolar macrophages as the first line of response in the host. A previous study [15] showed that a sublethal *P*. *marneffei* infection in BALB/c mice triggered a protective T helper lymphocyte Th1 immune response, as well as interferon-gamma (IFN-γ) expression to activate fungicidal macrophages through the L-arginine-dependent nitric oxide pathway. This indicates that the host immune response against *P*. *marneffei* infection is mainly mediated by macrophages and T-lymphocytes [15, 16]. In terms of the immune response, macrophage polarization is phenotypically and functionally plastic in order to respond to cytokine and fungus-sensing environments [17]. Functionally, pro-inflammatory macrophages are termed classically activated macrophages (M1), whereas those that inhibit inflammation and enhance tissue repair are termed alternatively activated macrophages (M2) [18]. M2 macrophages are further subdivided as M2a (after exposure to IL-4 or IL-13), M2b (immune complexes in combination with IL-1β or lipopolysaccharide [LPS]), and M2c (IL-10, transforming growth factor-beta [TGF-β] or glucocorticoids) [19, 20]. M1 cells can express iNOS to produce NO, and secrete significant amounts of pro-inflammatory cytokines, such as TNF-α and IL-12. M2 macrophages are characterized by expression of the enzyme Arg1, which hydrolyzes Arg1 to ornithine and urea, which are important for cellular proliferation and tissue repair [21]. M2a cells do not express iNOS, but express high levels of Arg1 and low levels of IL-10 [19]. Arg1 and iNOS enzymes can be used to ascertain the pathway of macrophage activation in rodents [22]. Our current study only focused on M1 and M2a macrophages, both of which are associated with antifungal responses [11, 23, 24]. Macrophages can be directed towards the M1 phenotype by Th1-type cytokines such as IFN-γ, or towards the M2 phenotype by Th2-type cytokines such as IL-4, IL-10, or IL-13 [19, 25]. These M1 and M2 macrophages will promote Th1 and Th2-induced host immune responses, respectively, however, Th1 and Th2 cytokines (e.g., IFN-γ or IL-4) can also down-regulate M2 and M1 activity for appropriate immune responses against pathogens [18]. Most research considering macrophage polarization states that result in anti-fungal activity has the potential to provide a novel approach for the treatment of fungal infections. The stimulation of M1 macrophage activation and/or the prevention of M2 macrophage activation have the potential to provide protection against fungal infections, including *C. neoformans*, and *A. fumigates* [11, 24]. However, enhancing M2a polarization, have a protective role in defense against *Pneumocystis* infections [23].

*P. marneffei* cytoplasmic yeast antigen (CYA) is prepared from *P. marneffei* yeast cells and 61-, 54-, and 50-kDa antigens purified from CYA can be used either singly or in combination to detect antibody responses in a large percentage of individuals infected with *P. marneffei* [26]. These antigens have a strong homology (87% identity) with the antioxidant enzyme catalase. Catalase antigens are known to be produced by a number of pathogenic fungi including *Histoplasma capsulatum* and *Aspergillus* fumigates and may play a role in inducing immune responses [27, 28]. These studies demonstrated that the CYA could have the ability to activate an immune response.

Alveolar macrophage polarization states during *P. marneffei* infection are unknown. Further, it has not been reported whether CYA crude antigen can stimulate alveolar macrophages. In this study, we utilized a mouse model to investigate alveolar macrophage polarization after *P*. *marneffei* infection, as well as the possible role of CYA in alveolar macrophage polarization.

## Results

### Isolation and identification of the GXHCBR *P*. *marneffei* strain

**Fig. 1:**
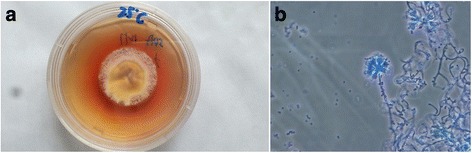
Identification of *P*. *marneffei* strain by culture and Lactophenol cotton blue staining. **a**, *P*. *marneffei* strain was cultured in potato dextrose agar: A unique characteristic of *P*. *marneffei* mold is that it can produce a soluble red pigment that diffuses into the agar at 25 °C. **b,** Microscopic examination revealed hyaline filamentous forms with branches, sometimes with chains of smooth conidia giving the appearance of a brush compatible with *P. marneffei* by Lactophenol cotton blue staining (×400)

The GXHCBR *P*. *marneffei* strain was isolated from lung, liver, and spleen of the bamboo rat. The *P*. *marneffei* strain was then cultured in potato dextrose agar and grew as a mold at 25 °C. A unique characteristic of *P*. *marneffei* mold is that it can produce a soluble red pigment that diffuses into the agar (Fig. 1a). A typical mold was observed as hyaline filamentous forms with branches, sometimes with chains of smooth conidia giving the appearance of a brush compatible with *P*. *marneffei* after Lactophenol cotton blue staining (Fig. 1b). Also these fungi were identified by gold standard DNA sequencing of the fungal internal transcribed spacer (ITS) region (Additional file 1).

### Confirmation in an animal model and alveolar macrophages

**Fig. 2:**
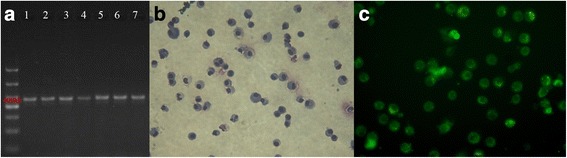
Confirmation of Animal model and alveolar macrophages. **a**, The MP1 PCR products were amplified from the mold on the plate of infected mice. Lane 1, PCR products (422 bp) of MP1gene from the GXHCBR *P*. *marneffei* strain. Lane 2–4, PCR products (422 bp) of the MP1gene from the molds of two weeks post-infected mice. Lane 5–7, PCR products (422 bp) of the MP1gene from the molds of four weeks post-infected mice. **b,** Microscopic examination shows round cells with a large and dark nucleus by Wright’s staining (× 400). **c,** Immunofluorescence for CD68 expression in alveolar macrophages (× 400)

Lung tissues were resected from *P*. *marneffei* infected mice and cultured to isolate *P*. *marneffei* in potato dextrose agar. This formed typical molds in 5–7 days. *P*. *marneffei*-specific MP1 PCR data confirmed that mold on the plate was the *P*. *marneffei* pathogen (Fig. 2a). Alveolar macrophages from infected mice were cultured and stained with Wright’s stain, which showed round cells with large and dark nuclei under a light microscope (Fig. 2b). Alveolar macrophage identification was by immunostaining for the macrophage cell marker CD68. CD68+ macrophages were identified by their green fluorescent (Fig. 2c).

### Isolation and confirmation of *P*. *marneffei* CYA

**Fig. 3:**
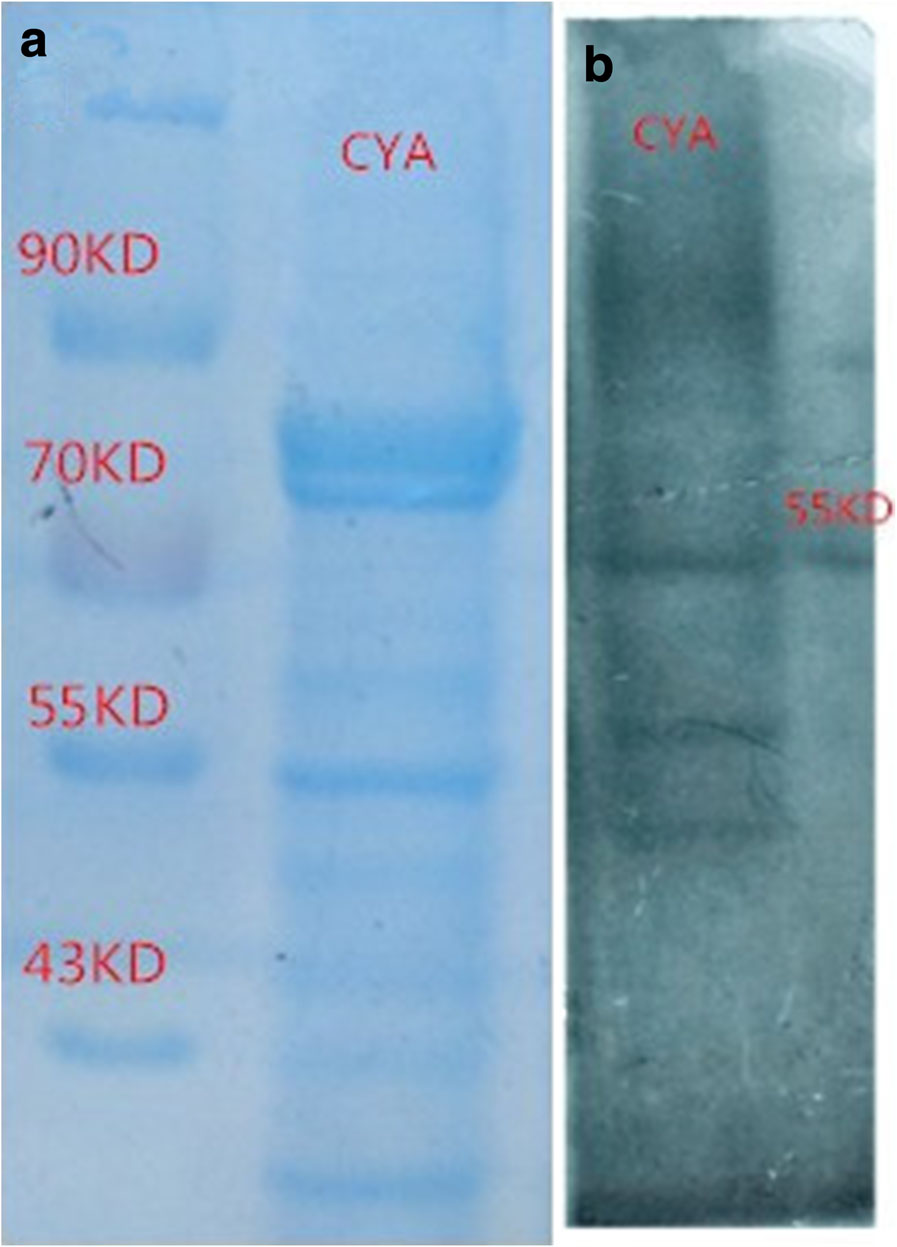
Identification of CYA by Western blot. **a**, The SDS-PAGE gel was prepared to separate CYA samples and stained with coomassie brilliant blue. **b**, CYA samples were assayed by Western blot analysis using sera from a HIV negative individual with *P*. *marneffei* infection. The most prominent band was identified as the 54-kDa antigen

CYA was extracted from the GXHCBR strain of *P*. *marneffei* in the yeast phase. We then confirmed these preparations to be CYA using Western blot. The CYA extracts were subjected to sodium dodecyl sulfate-polyacrylamide gel electrophoresis (SDS-PAGE) and the gels stained with Coomassie brilliant blue with the relative molecular masses of the protein bands observed (Fig. 3a). Proteins were subjected to Western blot analysis using sera from a HIV negative individual with *P*. *marneffei* infection and the blots identified a 54-kDa antigen (Fig. 3b). Following that, we performed matrix-assisted laser desorption/ionization time of flight mass spectrometry (MALDI-TOF MS) analysis of these 54-kDa antigens with Mascot software and the national center of biotechnology information (NCBI) non-redundant database from the *P. marneffei* strain (ATCC 18224 / CBS 334.59 / QM 7333). The 54-kDa antigens had a strong homology (81.82% identity) with the Bifunctional catalase-peroxidase Cat2 (score > 1000, Table 1).

**Table 1:** Proteomic analysis of the 54-kDa antigen

Antigen source	Accession no.	Protein name and species	Score	Coverage	MW [kDa]/pI
GXHCBR	B6QK96	Bifunctional catalase-peroxidase Cat2; Penicillium marneffei (strain ATCC 18224/CBS 334.59 / QM 7333)	1192.13	81.82	82.3/6.95

### Differential expression of M1 and M2a-related cytokines and key enzymes in alveolar macrophages

**Fig. 4:**
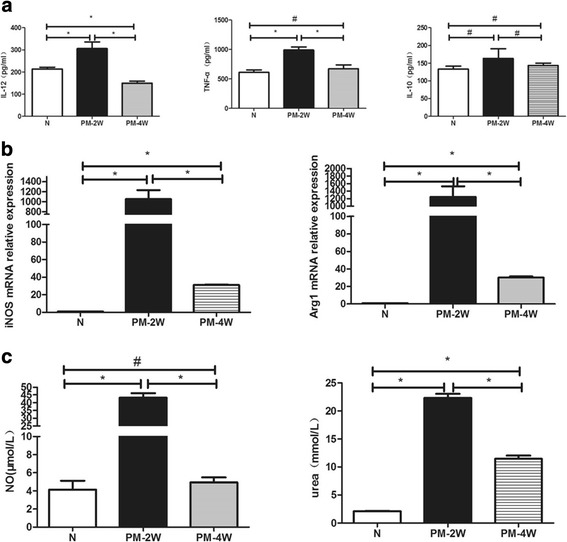
M1 and M2a-related cytokines and key enzymes expression after *P*. *marneffei* infection. **a**, ELISA. Supernatant from alveolar macrophages of normal control (N) and *P*. *marneffei* infected (PM) mice were collected and analyzed for IL-12, TNF-α and IL-10 (M1 and M2a-related cytokines) levels. The data are represented as mean ± SD (*N* = 4) and analyzed using ANOVA. *P < 0.05 and ^#^ *P* > 0.05. **b,** qRT-PCR. Alveolar macrophages were collected from normal control (N) and *P*. *marneffei* infected (PM) mice and subjected to qRT-PCR for iNOS and Arg1 gene (M1 and M2a-related key enzymes) expression. The data are expressed as mean ± SD (*N* = 5) and analyzed using ANOVA. *P < 0.05. **c,** Griess and arginase activity assays. Supernatant from alveolar macrophages of normal control (N) and *P*. *marneffei* infected (PM) mice were subjected to NO production using Griess activity assays, and alveolar macrophages were subjected to Urea production using arginase activity assays, respectively. The data are expressed as mean ± SD (*N* = 5) and analyzed using ANOVA. *P < 0.05 and ^#^ *P* > 0.05

First, we prepared the conditioned media from alveolar macrophage cultures and analyzed for cytokines levels using ELISA. The data showed that levels of IL-12 and TNF-α were significantly higher in the conditioned media from alveolar macrophages two weeks post-infection than both the control group and the fourth week post-infection group. Conversely, there was no statistical difference in IL-10 levels among these groups. The above indicators in the fourth week post-infection group were decreased significantly when compare with the second week post-infection group (Fig. 4a). Next, we analyzed key enzymes from alveolar macrophages using qRT-PCR, Griess and arginase activity assays. Moreover, similar trends were observed in the relative levels of iNOS and Arg1 mRNA and their activity products NO and urea (Fig. 4 b & c). These data indicate that alveolar macrophage M1/M2a subtypes were present in host defense against acute *P*. *marneffei* infection.

#### Effects of CYA on alveolar macrophage polarization

**Fig. 5:**
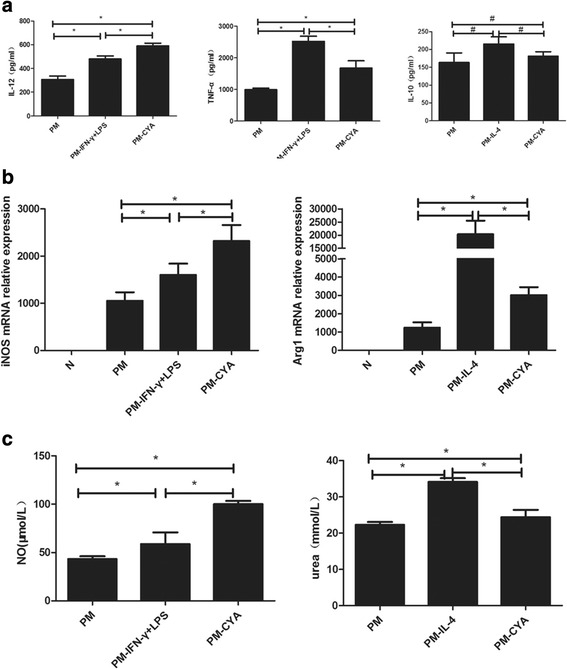
Effect of CYA on alveolar macrophage polarization in two weeks *P*. *marneffei* infected mice. Alveolar macrophages from two weeks *P*. *marneffei* infected (PM) mice were purified and treated as described in Methods. No stimuli served as a negative control (PM group), IFN-γ + LPS were included as the M1 positive control (PM- IFN-γ + LPS group), IL-4 was included as the M2a positive control (PM-IL-4 group) and CYA was included as an experimental group (PM-CYA group). **a,** ELISA. The supernatant from alveolar macrophages with different treatments were collected and analyzed for IL-12, TNF-α and IL-10 (M1 and M2a-related cytokines) levels. The data are represented as Mean ± SD (*N* = 4) and analyzed using ANOVA. *P < 0.05 and ^#^ *P* > 0.05. **b,** qRT-PCR. Alveolar macrophages after different treatments were collected and subjected to qRT-PCR for iNOS and Arg1 gene (M1 and M2a-related key enzymes) expression. The data are expressed as mean ± SD (*N* = 5) and analyzed using ANOVA. *P < 0.05. **c,** Griess and arginase activity assays. Alveolar macrophages and conditioned media were collected and subjected to Urea and NO production using arginase and Griess activity assays, respectively. The data are expressed as mean ± SD (*N* = 5) and analyzed using ANOVA. *P < 0.05 and ^#^ *P* > 0.05

**Fig. 6:**
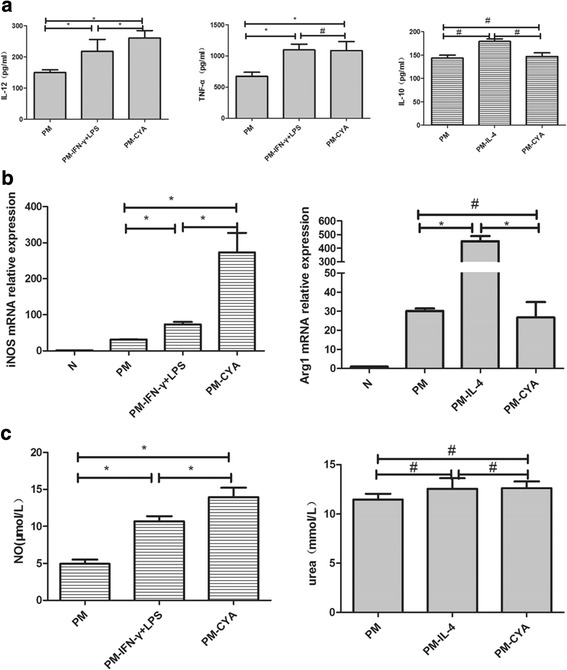
Effect of CYA on alveolar macrophage polarization in four weeks *P*. *marneffei* infected mice. Alveolar macrophages from four weeks *P*. *marneffei* infected (PM) mice were purified and treated as described above. **a**, ELISA. The supernatant from alveolar macrophages with different treatments were collected and analyzed for IL-12, TNF-α and IL-10 (M1 and M2a-related cytokines) levels. The data are represented as Mean ± SD (*N* = 4) and analyzed using ANOVA. *P < 0.05 and ^#^ *P* > 0.05. **b**, qRT-PCR. Alveolar macrophages from infected mice were treated with different agents and collected and subjected to qRT-PCR for iNOS and Arg1 gene (M1 and M2a-related key enzymes) expression. The data are expressed as mean ± SD (*N* = 5) and analyzed using ANOVA. *P < 0.05 and ^#^ *P* > 0.05. **c,** Griess and arginase activity assays. Alveolar macrophages and conditioned media were collected and subjected to Urea and NO production using arginase and Griess activity assays, respectively. The data are expressed as mean ± SD (*N* = 5) and analyzed using ANOVA. *P < 0.05 and ^#^ *P* > 0.05

To investigate the effects of CYA on alveolar macrophage polarization on *P*. *marneffei* infected mice, we treated alveolar macrophages with no stimuli as negative control (PM group, PM is an abbreviation of *P. marneffei*), IFN-γ + LPS as the M1 positive control (PM-IFN-γ + LPS group), IL-4 as the M2a positive control (PM- IL-4 group) and CYA (PM-CYA group), respectively. We found that during the second week post-infection, CYA stimulation significantly increased expression of IL-12, iNOS mRNA and NO when compared to the negative control group and M1 positive control group, and significantly increased expression of TNF-α when compared to the negative control group. CYA stimulation also significantly increased expression of Arg1 mRNA and urea when compared to the negative control group, however, this was decreased in comparison with the M2a positive group. Similarly, no significant change was found in IL-10 levels among all groups (Fig. 5). During the fourth week post-infection, similar trends were observed whereby CYA stimulation significantly increased expression of M1-related cytokines and key enzymes when compared to the negative control group and M1 positive control group. However, CYA stimulation could not enhance the expression of M2a-related cytokines and key enzymes. In comparison with two weeks post-infection, the above markers significantly decreased in the four weeks post-infection group (Fig. 6). The results suggested that CYA stimulation enhanced M1 and M2a macrophage polarization, which was consistent with the results for *P*. *marneffei* infection. Also, CYA stimulation preferentially enhanced expression of the M1-related cytokines and key enzymes in *P*. *marneffei* infected mice.

## Discussion

Systemic mycosis caused by *P. marneffei* is known to be endemic in South and Southeast Asia. It is often associated with immunocompromised patients, although, in recent years it is increasingly observed in individuals without HIV infection. The different clinical manifestations of *P. marneffei* infection depend on the immune status of the host. The alveolar macrophage is the first line of defense against *P*. *marneffei*. The objective of this study was to dissect the polarization states of alveolar macrophages on the regulation of mouse immune responses against *P*. *marneffei* infection. We found that two weeks of infection with *P*. *marneffei* significantly induced mouse immune responses including presentation of M1 and M2a macrophages in lung tissues. This decreased significantly in the fourth week of infection. We further confirmed our in vivo data using CYA. During *P. marneffei* infection, the polarization tendency of alveolar macrophages induced by CYA was consistent with the results for *P*. *marneffei* infection and CYA stimulation preferentially induced the M1 subtype. The data from the current study demonstrated that M1/M2a macrophages were present in lung tissues in response to acute *P*. *marneffei* infection, and that CYA could be responsible for *P*. *marneffei* infection induced host immune responses in mice, enhancing M1 subtype macrophages.

*P. marneffei* infection is endemic in Southeast Asia and China. However, the transmission route of *P. marneffei* has not yet been verified by large epidemiological investigations. Inhalation of conidia from the environment is thought to be the most likely transmission route. Thus, in the current study, we produced a mouse model of *P. marneffei* infection through intranasal administration. Furthermore, to identify *P. marneffei* infection, we utilized PCR to amplify the MP1 gene, which is a direct indicator for clinical diagnosis of *P. marneffei* infection according to a previous study [29]. In our current study, all animals were confirmed to be positive for the MP1 gene and *P. marneffei* culture, indicating that they were all successfully infected by *P. marneffei*.

It has been reported that *P*. *marneffei* is quickly removed by the spleen because the spleen expresses a high level of type 1 cytokines (IL-12 and INF-γ) as well as NO, which plays a crucial role in host defense against *P*. *marneffei* [15, 30]. In our current study, the level of M1-related iNOS/NO, TNF-α, IL-12, as well as M2a-related Arg1/urea were highly induced in alveolar macrophages by acute *P*. *marneffei* infection. With the progression of infection, their expression was significantly reduced. It has been demonstrated that macrophages can rapidly and uniformly reverse their polarization phenotype in response to different microenvironments and lose or regain their fungicidal function [24]. Moreover, during dimorphic fungus *Sporothrix schenckii* infection, cell wall peptide-polysaccharide (PPC) of this pathogen was able to induce IL-12 production, which was consistent with increases in NO production over the same period of time [31]. In our experiment, CYA prepared from *P. marneffei* yeast cells were separated by SDSPAGE and analyzed by Western blot with sera samples from *P. marneffei*-affected individuals, and recognized a series of molecular mass bands. The most important band was at approximately 54-kDa. We performed MALDI-TOF MS analysis of these 54-kDa antigens, which had a strong homology (81.82% identity) with the Bifunctional catalase-peroxidase Cat2. This was the first investigation into CYA as stimuli on alveolar macrophage polarization. During *P. marneffei* infection, we found that CYA stimulation could enhance M1-related iNOS mRNA/NO, TNF-α, and IL-12 expression, especially in the second week post-infection. CYA stimulation also could enhance M2a-related Arg1 mRNA /urea expression. There was no statistical difference in IL-10 levels among all groups. It was consistent with the results for *P*. *marneffei* infection and CYA stimulation had a tendency toward increased in M1 subtype macrophages. Our data indicate that CYA could mimic *P*. *marneffei* to induce a host immune response and enhance in M1 subtype macrophage polarization, which in turn promotes macrophages to develop enhanced microbicide activity.

Therapeutics targeting modifications of the host response rather than the pathogen would limit selective pressure on the microbe that can lead to drug resistance and increased virulence [11]. Our current study provides proof-of-principle by exploring mouse immune responses against *P*. *marneffei* infection. Future studies could develop anti-CYA vaccines to prevent *P*. *marneffei* infection in humans and animals.

## Conclusions

Macrophages play a crucial role in the host anti-*P*. *marneffei* infection response. Our current study demonstrates for the first time to the best of our knowledge that M1/M2a subtype macrophages were present in host defense against acute *P*. *marneffei* infection, while CYA could mimic *P*. *marneffei* to induce a host immune response and had a tendency to enhance M1 subtype macrophages. Thus, further studies are needed to investigate the enhancement of host anti-*P*. *marneffei* immune responses and to provide novel ideas for prevention of *P*. *marneffei*-infection.

## Methods

### *P*. *marneffei* strain and conidia preparation

The GXHCBR *P*. *marneffei* strain was isolated from the organs of the bamboo rat (the lungs, liver, or spleen) from Hechi city, Guangxi Province in Southern China. These fungi were cultured in potato dextrose Agar (PDA) medium (5.0 g potato extract, 20.0 g dextrose, 20.0 g agar, and 0.1 g chloromycetin) at 25 °C for 7 to 10 days, and identified by morphology and PCR analysis of ITS rDNA sequences. PCR amplification was performed using a Lightcycle 480 system (Roche Applied Science, Basel, Swiss) with primers (ITS1, 5′-TCCGTAGGTGAACCTGCGG-3′; ITS4, 5′-TCCTCCGCTTATTGATATGC-3′) at conditions of 95 °C for 5 min, followed by 30 cycles of 94 °C for 30s, 52 °C for 30s, and 72 °C for 1 min, and a final extension at 72 °C for 10 min.

The conidia were collected by flooding the culture surface with phosphate buffered saline (PBS) and counted using a hemocytometer. They were then suspended at a concentration of 5 × 10^7^ colony-forming units (CFU)/ml.

### Preparation and confirmation of *P*. *marneffei* cytoplasmic yeast antigen (CYA)

*P*. *marneffei* CYA was extracted according to a published study with some modifications [26] . Briefly, the GXHCBR *P*. *marneffei* strains were cultured in the yeast phase for 7 days at 37 °C on brain/heart infusion medium (BHIM) broth (10 g tryptone, 5 g sodium chloride, 2.5 g disodium hydrogen phosphate, 2 g dextrose, and 500 ml beef heart infusion). The medium was then refreshed with BHIM medium and cultured for an additional 7 days. We then harvested the yeast form of the fungus through centrifugation to remove the supernatant. CYA was prepared by mixing packed yeast cells with an equal volume of 0.5-mm glass Ballotini beads in PBS. The mixture was then disrupted by sonication (UP100H, Hielscher, Teltow, Germany) and the insoluble debris was removed by centrifugation at 7000 g for 20 min at 4 °C. The cytoplasmic antigen solution was then passed through a 0.45 μm filter (Corning Inc., Corning, NY, USA). The protein concentration of *P*. *marneffei* extracts was then determined by the bicinchoninic acid (BCA) protein assay kit (Cat #P0018A, Beyotime, Haimen, China), and the extracts were stored at −80 °C until use. We then confirmed these preparations to be CYA using Western blot. The extracts were subjected to sodium dodecyl sulfate-polyacrylamide gel electrophoresis (SDS-PAGE) in 10% SDS-PAGE gels to detect molecular weight, and Western blotting to detect antigens. After SDS-PAGE, the gels were stained with Coomassie brilliant blue and the relative molecular masses of the protein bands were compared to the prestained protein ladder (Cat #26617, Thermo Scientific, Rockford, IL, USA). After proteins transferred to the nitrocellulose membrane, the membranes were subjected to Western blot analysis using sera from a HIV negative individual with *P*. *marneffei* infection at a dilution of 1:250. Following that, goat anti-human immunoglobulin G (IgG) conjugated with peroxidase was added to the membrane at a dilution of 1:2000 and the blots were visualized with enhanced chemiluminescence (Cat #P0018A, Beyotime) and quantified using the EQ Imaging System (Bio-Rad Laboratories, Inc., Hercules, CA, USA).

### MALDI-TOF MS analysis

54-kDa protein spots of GXHCBR *P*. *marneffei* strains were manually excised from SDS-PAGE gels, subjected to in situ digestion with trypsin, and then analyzed using a 4800 Plus (Applied Biosystems, Foster city, CA, USA) according to manufacturer’s protocol.

### Animals model preparation and confirmation

A total of 150 five to six-week old male BALB/c mice weighing 20 to 24 g were purchased from the Laboratory Animal Centre of Guangxi Medical University and were randomly assigned to either the *P. marneffei* uninfected group with 30 mice, two weeks *P. marneffei*-infected group, or four weeks *P. marneffei*-infected group with 60 mice in each group. Mice were infected with the GXHCBR *P*. *marneffei* strain according to a published study with some modifications [16]. BALB/c mice were narcotized with diethyl ether and then a sub-lethal suspension of *P*. *marneffei* conidia (2.5 × 10^6^ conidia per mouse in 50 μl PBS) was slowly dripped into the nose of each mouse and raised for two or four weeks. The negative control mice were without any treatment. The mice received sterilized food and water at the laboratory animal care center. Animal care was in accordance with national and institutional policies for animal health and well-being. The experimental protocol was approved by the Animal Care and Welfare Committee of Guangxi Medical University.

Anesthetized mice were sacrificed at two and four weeks after *P*. *marneffei* infection and dissected to evaluate *P*. *marneffei* growth. Part of the lung tissues were removed, plated and cultured in the PDA medium at 25 °C. The molds on the plate were collected for *P*. *marneffei* specific MP1 PCR amplification with primers (5′-CCACGAACTCGCCGACATTTC-3′ and 5′-CAGAGGAACGACAGGAACGGA-3′ to generate a PCR product of 422 bp) under the conditions of 95 °C for 5 min and 30 cycles of 94 °C for 30s, 55 °C for 30s, and 72 °C for 45 s, followed by a final extension of 72 °C for 10 min. PCR products were analyzed by 2% agarose gel electrophoresis and images were analyzed by Gel Doc EQ Imaging System (Bio-Rad Laboratories, Inc.).

### Alveolar macrophage isolation and culture

Alveolar macrophages were isolated from bronchoalveolar lavage fluid (BALF) by centrifugation at 300 g for 15 min at 4 °C. The cells were then resuspended in roswell park memorial institute 1640 (RPMI-1640) medium (Gibco) supplemented with 100 U/mL penicillin, 100 μg/mL streptomycin, referred to here as RPMI-1640 complete (RPMI-1640C) medium at a density of 1 × 10^6^/well and seeded into 12-well plates to allow macrophages to adhere. Macrophages were purified by adherence for 2.5 h at 37 °C in a 5% carbon dioxide (CO2) incubator and yielded >95% purity. Then macrophage were stained with Wright stain and immunostaining for CD68 and viewed under an inverted microscope.

The adhered alveolar macrophages were then divided into five groups: i) normal group (N group) from uninfected mice were cultured in 1 ml of RPMI-1640C medium alone; ii) *P*. *marneffei* infected group (PM group) from infected mice were incubated in 1 ml of RPMI-1640C medium; iii) M1 positive control group (PM-IFN-γ + LPS group) from infected mice were incubated in 1 ml of RPMI-1640C medium containing 100 U/ml IFN-γ and 5 ng/ml lipopolysaccharide (LPS); iv) M2a positive control group (PM-IL-4 group) from infected mice were incubated in 1 ml of RPMI-1640C medium containing 100 ng/ml IL-4; and v) the experimental group (PM-CYA group) from infected mice incubated in 1 ml of RPMI-1640C medium containing 50 μg/ml CYA. All of groups were cultured at 37 °C in 5% CO2 for 24 h. At the end of each experiment, the conditioned medium of each group were collected and assayed for nitric oxide and cytokine level, while alveolar macrophages were collected for qRT-PCR and Arg1 activity assay.

### ELISA detection of cytokine levels

Levels of IL-12, TNF-α and IL-10 in alveolar macrophage-conditioned medium were measured using ELISA kits (Cat #CSB-E07360m, #CSB-E04594m, and #CSB-E04741m; CUSABIO, Wuhan, China) according to the manufacturer’s instructions.

### qRT-PCR detection of iNOS, Arg1mRNA levels

Total RNA from alveolar macrophages were isolated using the TRIzol reagent (Invitrogen, Carlsbad, CA, USA) to identify iNOS and Arg1 mRNA. RNA samples (1 μg each) were reverse transcribed into cDNA using a reverse transcription kit (Cat #RR047A; Takara, Dalian, China). The qPCR primers were designed by using Primer Express Software and synthesized by Takara (see Table 2). qPCR was carried out using SYBR Green Master (ROX) reagents (Cat #RR820A; Takara) in the ABI Stepone Plus (ABI Biosystems). The reaction was initiated for 95 °C for 30 s, followed by 40 cycles of 95 °C for 5 s and 60 °C for 30 s. Data were normalized to GAPDH (reference genes) and calculated using the 2-ΔΔCt method.

**Table 2:** Primer sequences used for qPCR

Gene	Sequences	Product size (bp)
iNOS	5′-CAAGCACATTTGGGAATGGAGA-3′ 5′-CAGAACTGAGGGTACATGCTGGAG-3’	136
Arg1	5’-AGCTCTGGGAATCTGCATGG-3′ 5′-ATGTACACGATGTCTTTGGCAGATA-3’	125
GAPDH	5’-TGTGTCCGTCGTGGATCTGA-3′ 5′-TTGCTGTTGAAGTCGCAGGAG-3’	150
T-bet	5’-CATGGAGAACGGAGAATGGA-3′ 5′-TGGACAGGGGAAGAGAGCA-3’	118
GATA-3	5’-GGATGTAAGTCGAGGCCCAAG-3′ 5′-ATTGCAAAGGTAGTGCCCGGTA-3’	117
β-actin	5’-CATCCGTAAAGACCTCTATGCCAAC-3′ 5′-ATGGAGCCACCGATCCACA-3’	171

### Griess assay to detect NO level

50 μl aliquots of alveolar macrophage-conditioned medium were mixed with 50 μl of Griess reagent (Applygen Technologies Inc., Beijing, China) and incubated for 10 min at the room temperature in the dark. The colorimetric reaction was then measured at 540 nm using a Mutiskan Go microplate reader (Thermo Scientific, Rockford, IL, USA).

### Arginase activity assay to detect urea level

Arginase activity was measured using a method described by Corraliza et al. [32] . Briefly, 1 × 10^6^ alveolar macrophages were lysed with 50 μl of 0.1% Triton X-100 for 30 min and then added to 50 μl of 50 mmol/L Tris-HCl/10 mmol/L Cl2Mn·4H2O (pH 7.5) and incubated at 55 °C for 10 min. L-arginine hydrolysis was carried out by incubating with 25 μl of 0.5 M L-arginine (pH 9.7) at 37 °C for 60 min. The reaction was then stopped with 400 μl of stop solution [H2SO4 (96%)/H3PO4 (85%)/H2O (1:3:7, *v*/v/v)] and 25 μl of 9% of 2-isonitrosopropiophenone. The reactions were incubated at 100 °C for 45 min and 100 μl of each sample was analyzed using a microplate reader at 540 nm. A standard curve was generated from urea solutions (0–20 mM), which were used to determine the final concentrations.

### Statistical analysis

The data were summarized as the mean ± standard deviation (SD) of at least three-independent experiments. Statistical analyses were performed using SPSS16.0 (SPSS, Chicago, IL, USA) and the two-sample t-test was carried out to compare difference between groups and one-way analysis of variance (ANOVA) for multiple comparisons. The probability level ≤ 0.05 was considered as statistically significant.

## Additional files

Additional file 1: Title of data- Sequences of *P. marneffei* strains using PCR and the sequence of PCR product. Description of data- The GXHCBR *P*. *marneffei* strains were identified by gold standard DNA sequencing of the fungal ITS region using PCR and the sequence of PCR product. (DOCX 12 kb)

Additional file 2: Title of data-raw data. Description of data- The raw data of Fig. 4, Fig. 5 and Fig. 6, please see Tables 1, 2, and 3, respectively. (DOCX 27 kb)

## Abbreviations

ANOVA: One-way analysis of variance
Arg1: Arginase1
BALF: Bronchoalveolar lavage fluid
BCA: Bicinchoninic acid
BHIM: Brain/heart infusion medium
CFU: Colony-forming units
CO2: Carbon dioxide
CYA: Cytoplasmic yeast antigen
ELISA: Enzyme-linked immunosorbent;
HIV: Human immunodeficiency virus
IFN-γ: Interferon- gamma
IgG: Immunoglobulin G
IL-12: Interleukin-12
iNOS: Inducible nitric oxide synthase
ITS: Internal transcribed spacer
LPS: Lipopolysaccharide
M1: Classically activated macrophage
M2a: Alternatively activated macrophage
MALDI-TOF MS: Matrix-assisted laser desorption/ionization time of flight mass spectrometry
N: Normal
NCBI: National center of biotechnology information
NO: Nitric oxide
PBS: Phosphate buffered saline
PDA: Potato dextrose Agar
PM: *P. marneffei*
PPC: Peptide-polysaccharide
qRT-PCR: Quantitative real-time PCR
RPMI 1640: Roswell park memorial institute 1640
RPMI-1640C: RPMI-1640 complete
SD: Standard deviation
SDS-PAGE: Sodium dodecyl sulfate-polyacrylamide gel electrophoresis
TGF-β: Transforming growth factor-beta
TNF-α: Tumor necrosis factor-alpha
