# Supplementary material for: Acute Penicillium marneffei infection stimulates host M1/M2a macrophages polarization in BALB/C mice
Source: BMC Microbiol. 2017 Aug 18;17:177. doi: 10.1186/s12866-017-1086-3 (PMC5563047; doi:10.1186/s12866-017-1086-3)
Supplement: Supplementary file 1 — Title of data- Sequences of P. marneffei strains using PCR and the sequence of PCR product. Description of data- The GXHCBR P. marneffei strains were identified by gold standard DNA sequencing of the fungal ITS region using PCR and the sequence of PCR product. (DOCX 12 kb) [file 12866_2017_1086_MOESM1_ESM.docx]

Sequences of *P*. marneffei strains using PCR and the sequence of PCR product:

CCTCGCGGCCAACCTCCCACCCTTGTCTCTATACACCTGTTG

CTTTGGCGGGCCCACCGGGGCCACCCGGTCGCCGGGGGACG

TTTGTCCCCGGGCCCGCGCCCGCCGAAGCGCCCTGTGAACCC

TGATGAAGATGGACTGTCTGAGTACCATGAAAATTGTCAAA

ACTTTCAACAATGGATCTCTTGGTTCCGGCATCGATGAAGAA

CGCAGCGAAATGCGATAAGTAATGTGAATTGCAGAATTCCG

TGAATCATCGAATCTTTGAACGCACATTGCGCCCCCTGGCAT

TCCGGGGGGCATGCCTGTCCGAGCGTCATTTCTGCCCTCAAG

CACGGCTTGTGTGTTGGGTGTGGTCCCTCCGGGGACCTGCCC

GAAAGGCAGCGGCGACGTCCGTCTGGTCCTCGAGCGTATGG

GGCTCTGTCACTCGCTCGGGAAGGACCTGCGGGGGTTGGTC

ACCACCATATTTACCACGGTTGACCTCGGATCAGGTAGGAGT

TACCCGCTGAACTTAAGCATATCTA
