# Supplementary material for: Acute Penicillium marneffei infection stimulates host M1/M2a macrophages polarization in BALB/C mice
Source: BMC Microbiol. 2017 Aug 18;17:177. doi: 10.1186/s12866-017-1086-3 (PMC5563047; doi:10.1186/s12866-017-1086-3)
Supplement: Supplementary file 2 — Title of data-raw data. Description of data- The raw data of Fig. 4, Fig. 5 and Fig. 6, please see Tables 1, 2, and 3, respectively. (DOCX 27 kb) [file 12866_2017_1086_MOESM2_ESM.docx]

Table 1 **M1 and M2a-related cytokines and key enzymes expression** **after *P. marneffei* infection.**

|  | N | PM-2W | PM-4W |
| --- | --- | --- | --- |
| IL-12(pg/ml) | 204.15 | 311.28 | 155.19 |
|  | 213.36 | 275.29 | 158.95 |
|  | 222.14 | 289.94 | 141.37 |
|  | 216.15 | 345.06 | 143.72 |
|  |  |  |  |
|  |  |  |  |
| TNF-α(pg/ml) | 593.57 | 1044.18 | 700.65 |
|  | 663.32 | 954.83 | 573.14 |
|  | 618.52 | 1022.81 | 729.23 |
|  | 575.17 | 931.81 | 678.58 |
|  |  |  |  |
|  |  |  |  |
| IL-10(pg/ml) | 138.79 | 197.82 | 135.29 |
|  | 135.37 | 167.59 | 149.2 |
|  | 121.41 | 132.1 | 140.32 |
|  | 137.85 | 155.12 | 148.71 |
|  |  |  |  |
|  |  |  |  |
|  |  |  |  |
| iNOSmRNA relative expression | 1 | 820.2283 | 30.65145 |
|  | 1 | 1169.595 | 31.46811 |
|  | 1 | 930.4423 | 31.50751 |
|  | 1 | 1077.076 | 30.22167 |
|  | 1 | 1269.419 | 31.54264 |
|  |  |  |  |
|  |  |  |  |
| Arg1mRNA relative expression | 1 | 827.4943 | 30.04835 |
|  | 1 | 1262.067 | 29.374 |
|  | 1 | 1148.365 | 28.66156 |
|  | 1 | 1549.22 | 31.85695 |
|  | 1 | 1452.349 | 30.84617 |
|  |  |  |  |
|  |  |  |  |
| NO（μmol/L) | 4.372 | 40.539 | 5.53 |
|  | 5.63 | 45.472 | 5.19 |
|  | 4.061 | 46.999 | 4.4 |
|  | 3.654 | 41.09 | 4.307 |
|  | 3 | 41.57 | 5.28 |
|  |  |  |  |
|  |  |  |  |
| urea（mmol/L) | 2.18 | 21.78 | 12.22 |
|  | 2.09 | 23.04 | 11.72 |
|  | 2.12 | 22.85 | 11.39 |
|  | 2.04 | 22.61 | 11.31 |
|  | 2.07 | 21.18 | 10.62 |

N: normal group (uninfected group); PM-2W: two weeks *P*. *marneffei* infected (PM) group; PM-4W: four weeks *P*. *marneffei* infected (PM) group

**Table 2 Effect of CYA on alveolar macrophage polarization in two weeks *P*. *marneffei* infected mice.**

|  | N | PM | PM-IFN-γ+LPS  or PM-IL-4 | PM-CYA |
| --- | --- | --- | --- | --- |
| IL-12(pg/ml) |  | 311.28 | 471.57 | 587.07 |
|  |  | 275.29 | 511.32 | 561.96 |
|  |  | 289.94 | 484.96 | 616.36 |
|  |  | 345.06 | 451.5 | 593.82 |
|  |  |  |  |  |
|  |  |  |  |  |
| TNF-γ(pg/ml) |  | 1044.18 | 2626.48 | 1515.66 |
|  |  | 954.83 | 2317.09 | 1506.05 |
|  |  | 1022.81 | 2668.6 | 1997.23 |
|  |  | 931.81 | 2461.28 | 1680.26 |
|  |  |  |  |  |
|  |  |  |  |  |
| IL-10(pg/ml) |  | 197.82 | 188.32 | 199.727 |
|  |  | 167.59 | 237.34 | 178.14 |
|  |  | 132.1 | 214.32 | 171.56 |
|  |  | 155.12 | 220.9 | 174.85 |
|  |  |  |  |  |
|  |  |  |  |  |
|  |  |  |  |  |
| iNOSmRNA relative expression | 1 | 820.2283 | 1658.082 | 2767.736 |
|  | 1 | 1169.595 | 1332.224 | 2559.334 |
|  | 1 | 930.4423 | 1973.755 | 2259.768 |
|  | 1 | 1077.076 | 1539.398 | 2014.885 |
|  | 1 | 1269.419 | 1509.113 | 2002.876 |
|  |  |  |  |  |
|  |  |  |  |  |
| Arg1mRNA relative expression | 1 | 827.4943 | 15434.11 | 3248.591 |
|  | 1 | 1262.067 | 15042.01 | 2582.358 |
|  | 1 | 1148.365 | 26959.26 | 3065.965 |
|  | 1 | 1549.22 | 20717.34 | 2585.805 |
|  | 1 | 1452.349 | 23546.41 | 3591.781 |
|  |  |  |  |  |
|  |  |  |  |  |
| NO（μmol/L) |  | 40.539 | 49.533 | 101.81 |
|  |  | 45.472 | 50.835 | 105.09 |
|  |  | 46.999 | 48.957 | 97.08 |
|  |  | 41.09 | 71.36 | 97.9 |
|  |  | 41.57 | 72.57 | 98.26 |
|  |  |  |  |  |
|  |  |  |  |  |
| urea（mmol/L) |  | 21.78 | 35.19 | 20.89 |
|  |  | 23.04 | 34.1 | 25.41 |
|  |  | 22.85 | 33.55 | 24.54 |
|  |  | 22.61 | 34.97 | 25 |
|  |  | 21.18 | 32.55 | 26.02 |

N: normal group (uninfected group); PM: two weeks *P. marneffei* infected (PM) group; PM-IFN-γ+LPS: M1 positive control group; PM-IL-4: M2a positive control group; PM-CYA: experimental group.

**Table3 Effect of CYA on alveolar macrophage polarization** **in four weeks *P*. *marneffei* infected mice.**

|  | N | PM | PM-IFN-γ+LPS  or PM-IL-4 | PM-CYA |
| --- | --- | --- | --- | --- |
| IL-12(pg/ml) |  | 155.19 | 193.27 | 248.09 |
|  |  | 158.95 | 193.69 | 294.12 |
|  |  | 141.37 | 210.43 | 260.65 |
|  |  | 143.72 | 274.14 | 238.76 |
|  |  |  |  |  |
|  |  |  |  |  |
| TNF-γ(pg/ml) |  | 700.65 | 1115.07 | 1044.18 |
|  |  | 573.14 | 1006.93 | 904.042 |
|  |  | 729.23 | 1044.18 | 1154.16 |
|  |  | 678.58 | 1216.89 | 1240.1 |
|  |  |  |  |  |
|  |  |  |  |  |
| IL-10(pg/ml) |  | 135.29 | 184.94 | 145.02 |
|  |  | 149.2 | 182.25 | 147.39 |
|  |  | 140.32 | 178.14 | 139.66 |
|  |  | 148.71 | 172.55 | 157.75 |
|  |  |  |  |  |
|  |  |  |  |  |
|  |  |  |  |  |
| iNOSmRNA relative expression | 1 | 30.65145 | 80.15723 | 218.9119 |
|  | 1 | 31.46811 | 67.79286 | 329.0815 |
|  | 1 | 31.50751 | 72.96378 | 235.2563 |
|  | 1 | 30.22167 | 80.35949 | 248.6651 |
|  | 1 | 31.54264 | 66.40296 | 334.1666 |
|  |  |  |  |  |
|  |  |  |  |  |
| Arg1mRNA relative expression | 1 | 30.04835 | 470.1613 | 23.87293 |
|  | 1 | 29.374 | 403.019 | 40.86316 |
|  | 1 | 28.66156 | 433.2062 | 22.90488 |
|  | 1 | 31.85695 | 503.334 | 21.1389 |
|  | 1 | 30.84617 | 449.1306 | 25.02981 |
|  |  |  |  |  |
|  |  |  |  |  |
| NO（μmol/L) |  | 5.53 | 10.49 | 12.33 |
|  |  | 5.19 | 11.06 | 12.79 |
|  |  | 4.4 | 11.32 | 14.56 |
|  |  | 4.307 | 9.57 | 15.28 |
|  |  | 5.28 | 10.96 | 14.75 |
|  |  |  |  |  |
|  |  |  |  |  |
| urea（mmol/L) |  | 12.22 | 12.11 | 12.32 |
|  |  | 11.72 | 12.08 | 12.59 |
|  |  | 11.39 | 11.34 | 11.83 |
|  |  | 11.31 | 14.1 | 13.68 |
|  |  | 10.62 | 13.15 | 12.66 |

N: normal group (uninfected group); PM: four weeks *P. marneffei* infected (PM) group; PM-IFN-γ+LPS: M1 positive control group; PM-IL-4: M2a positive control group; PM-CYA: experimental group.
